# Benchmarking health system performance across districts in Zambia: a systematic analysis of levels and trends in key maternal and child health interventions from 1990 to 2010

**DOI:** 10.1186/s12916-015-0308-5

**Published:** 2015-04-02

**Authors:** Katherine Ellicott Colson, Laura Dwyer-Lindgren, Tom Achoki, Nancy Fullman, Matthew Schneider, Peter Mulenga, Peter Hangoma, Marie Ng, Felix Masiye, Emmanuela Gakidou

**Affiliations:** University of California, Berkeley (UC Berkeley), Berkeley, CA USA; Institute for Health Metrics and Evaluation, University of Washington, Seattle, WA USA; Ministry of Health of Botswana, Gaborone, Botswana; USAID, Washington, DC USA; Clinton Health Access Initiative, Lusaka, Zambia; Department of Economics, University of Bergen, Bergen, Norway; Department of Economics, University of Zambia, Lusaka, Zambia

**Keywords:** Coverage, Indicators, Inequalities, Maternal and child health, Subnational benchmarking, Zambia

## Abstract

**Background:**

Achieving universal health coverage and reducing health inequalities are primary goals for an increasing number of health systems worldwide. Timely and accurate measurements of levels and trends in key health indicators at local levels are crucial to assess progress and identify drivers of success and areas that may be lagging behind.

**Methods:**

We generated estimates of 17 key maternal and child health indicators for Zambia’s 72 districts from 1990 to 2010 using surveys, censuses, and administrative data. We used a three-step statistical model involving spatial-temporal smoothing and Gaussian process regression. We generated estimates at the national level for each indicator by calculating the population-weighted mean of the district values and calculated composite coverage as the average of 10 priority interventions.

**Results:**

National estimates masked substantial variation across districts in the levels and trends of all indicators. Overall, composite coverage increased from 46% in 1990 to 73% in 2010, and most of this gain was attributable to the scale-up of malaria control interventions, pentavalent immunization, and exclusive breastfeeding. The scale-up of these interventions was relatively equitable across districts. In contrast, progress in routine services, including polio immunization, antenatal care, and skilled birth attendance, stagnated or declined and exhibited large disparities across districts. The absolute difference in composite coverage between the highest-performing and lowest-performing districts declined from 37 to 26 percentage points between 1990 and 2010, although considerable variation in composite coverage across districts persisted.

**Conclusions:**

Zambia has made marked progress in delivering maternal and child health interventions between 1990 and 2010; nevertheless, substantial variations across districts and interventions remained. Subnational benchmarking is important to identify these disparities, allowing policymakers to prioritize areas of greatest need. Analyses such as this one should be conducted regularly and feed directly into policy decisions in order to increase accountability at the local, regional, and national levels.

**Electronic supplementary material:**

The online version of this article (doi:10.1186/s12916-015-0308-5) contains supplementary material, which is available to authorized users.

## Background

Achievement of universal health coverage (UHC) is a primary goal for an increasing number of health systems worldwide and has been proposed as a key objective for the post-2015 development agenda [1]. UHC aims to provide all people with the high-quality health services they need without the risk of financial hardship from out-of-pocket expenses [2]. Included in UHC is the goal of reducing inequalities within countries, and this has led to an increased focus on within-country inequalities in low- and middle-income countries (LMICs) [3-6]. National gaps in UHC are closely related to inequalities in intervention coverage within countries [7,8]. While much progress has been made in reducing maternal and child mortality in the past two decades [9], many countries are lagging behind in the delivery of life-saving interventions and would benefit from intensified actions targeted to the worst-off and hardest-to-reach populations [10]. To inform these efforts, timely and accurate information is needed, and demand for the measurement of subnational coverage in maternal and child health (MCH) and for analysis of time trends in subnational inequality is increasing [11,12].

Information on subnational levels and trends in health in LMICs is limited but growing. To date, most studies and global monitoring systems have focused on within-country inequalities by wealth indices, education, gender, or urban residence [6-8,12-27]. While this literature has been immensely important in identifying strikingly large inequalities and informing policy in many countries, gathering information on variation by geographic subunits has been under-prioritized. Subnational benchmarking has been instrumental in decision-making in high-income countries [28-32], but explicit comparisons of performance across subunits over time remain uncommon in much of the developing world. The Countdown to 2015 group has routinely tracked progress and equity in MCH intervention coverage for 75 countries since 2005, but reports incorporating health measures at subnational geographic levels only began in 2010 [33]. Several studies, most commonly in India, have quantified coverage and outcomes at the regional [33-42] and first administrative levels [43-61]; however, most examine only one indicator, do not evaluate trends over time, or are not explicitly targeted to policymakers interested in local benchmarking for their countries. Even fewer studies have explored the geographic distributions for indicators below the first administrative level [62-76], which is arguably of greater policy relevance [77]. Mexico was the first LMIC country to implement subnational benchmarking of effective coverage [48,78-80] and to then have these data feed into policy decisions, demonstrating how locally-relevant data can be used to inform health policymaking.

In recent years, Zambia has demonstrated multi-stakeholder commitment to UHC and equity in health service delivery [81-84]. The country’s *National Health Strategic Plan 2011-2015* [82] diverges from previous plans in its emphasis on UHC and overall health system strengthening rather than vertical programs. Zambia has successfully scaled up many priority MCH interventions in the past two decades [82]. However, previous studies have focused on national trends and have not explored within-country inequalities. Accurate, timely, subnational information on intervention coverage is needed to benchmark progress and to pinpoint areas in need of targeted policy intervention. In this study, we use all available data to produce the first systematic assessment of levels and trends in the coverage of 17 MCH interventions, with estimates of uncertainty, across Zambia’s 72 districts from 1990 to 2010.

## Methods

### Data and indicator selection

We conducted several in-country meetings with major stakeholders in MCH to identify all available data sources with information on MCH and socioeconomic factors, including 20 household surveys, 3 population censuses, and 4 administrative data sources covering Zambia’s 72 districts (Additional file 1).

We identified 17 key indicators that are closely tied to child survival [85] and that could be estimated at the district level from the identified data sources: antenatal care (ANC, 1 and 4 visits); skilled birth attendance (SBA); immunization with Bacillus Calmette-Guérin (BCG); diphtheria-pertussis-tetanus (DPT); measles, polio, and pentavalent vaccines; exclusive breastfeeding (EBF); prevalence of underweight among children as a proxy for nutritional interventions [86]^a^; intermittent preventive therapy for malaria during pregnancy (IPTp, 1 and 2 doses); insecticide-treated net (ITN) ownership; ITN use by children under five; indoor residual spraying (IRS); the proportion of households that either owned at least one ITN or received IRS; and the proportion of children who either slept under an ITN the night before the survey or lived in a household that received IRS. Because several indicators were very similar, we report findings for 12 of these indicators in the main text and present results for the other five in Additional file 2. Other indicators of interest, including case management of childhood diarrhea and pneumonia, and artemisinin-based combination therapy for childhood malaria, could not be estimated due to sparse data at the district level. We did not include immunization coverage estimates constructed by pairing data on the number of doses administered with population figures, because, in contrast with survey-based estimates, such measures are often subject to significant numerator and denominator bias which are likely exacerbated at the district level [87]. Due to data availability, we restricted the analysis to the period from 1990 to 2010.

Our modeling strategy, described in more detail below, used the following covariates, identified based on previously established relationships [88-91] and strong correlations in our data: whether the household had electricity, sex of the head of household, household size, average years of education among women 15 to 44 years old, the use of improved wall materials in households, and the number of health facilities per capita in a district. The number of health facilities per capita was only available for the year 2006. Complete definitions of the indicators and covariates are provided in Table 1.

**Table 1:** **Definitions of indicators and covariates**

**Indicator or covariate (abbreviation)**	**Definition**	**Sources of data**
**Indicators**		
Antenatal care (ANC1, ANC4)	The proportion of women 15 to 49 years old who gave birth in the given year and had one/four or more antenatal visits attended by skilled personnel (doctor, nurse, midwife, or clinical officer) at a health facility during the corresponding pregnancy	DHS: 1992, 1996–7, 2001–2, 2007
		MIS: 2006, 2008, 2010, 2012
Skilled birth attendance	The proportion of women 15 to 49 years old who gave birth in the given year and delivered with a skilled birth attendant (a doctor, nurse, midwife, or clinical officer)	DHS: 1992, 1996–7, 2001–2, 2007
		MICS: 1999
Bacillus Calmette-Guérin immunization	The proportion of children under 5 years old who were vaccinated against tuberculosis in the given year	DHS: 1992, 1996–7, 2001–2, 2007
		LCMS: 1996, 1998, 2002–3, 2004–5, 2010
		MICS: 1999
Diphtheria-pertussis-tetanus immunization	The proportion of children 12 to 59 months old who received three doses of the diphtheria-pertussis-tetanus (DPT) vaccine in the given year	DHS: 1992, 1996–7, 2001–2, 2007
		LCMS: 1996, 1998, 2002–3, 2004–5, 2010
		MICS: 1999
Measles immunization	The proportion of children 12 to 59 months old who received measles vaccination in the given year	DHS: 1992, 1996–7, 2001–2, 2007
		LCMS: 1996, 1998, 2002–3, 2004–5, 2010
		MICS: 1999
Polio immunization	The proportion of children 12 to 59 months old who received three doses of the oral polio vaccine in the given year	DHS: 1992, 1996–7, 2001–2, 2007
		LCMS: 1996, 1998, 2002–3, 2004–5, 2010
		MICS: 1999
Pentavalent immunization	The proportion of children 12 to 59 months old who received three doses of the pentavalent vaccine, which includes protection against DPT, hepatitis B, and *Haemophilus influenzae* type b	DHS: 2007
		LCMS: 2010
Exclusive breastfeeding*	The proportion of children who were exclusively breastfed during their first 6 months after birth	DHS: 1992
		LCMS: 1996, 1998, 2003–3, 2004–5, 2010
Percentage of children not underweight	The proportion of children under 5 years old determined as not being underweight, defined as weighing two or more standard deviations below the international anthropometric reference population median of weight for age	DHS: 1992, 1996–7, 2001–2, 2007
		LCMS: 1996, 1998, 2002–3, 2004–5, 2006, 2010
Intermittent preventive therapy for malaria during pregnancy (IPTp1, IPTp2)	The proportion of women 15 to 49 years old who gave birth in the given year and who received at least one/two treatment doses of Fansidar (sulfadoxine/pyrimethamine) at antenatal care visits during the corresponding pregnancy	DHS: 2007
		MIS: 2006, 2008, 2010, 2012
Insecticide-treated net (ITN) ownership	The proportion of households that own at least one ITN	DHS: 2007
		HHCS: 2008
		MIS: 2006, 2008, 2010, 2012
		Netmark: 2000, 2004
		SBS: 2005, 2009
ITN use by children under five	The proportion of children under 5 years old who slept under an ITN the previous night	DHS: 2007
		HHCS: 2008
		MICS: 1999
		MIS: 2006, 2008, 2010, 2012
		Netmark: 2000, 2004
		SBS: 2005, 2009
Indoor residual spraying (IRS)	The proportion of households that were sprayed with an insecticide-based solution in the last 12 months	DHS: 2007
		HHCS: 2008
		MIS: 2006, 2008, 2010, 2012
		NMCC: 2005-2010
ITN ownership or IRS	The proportion of households that either own an ITN, or were sprayed with an insecticide-based solution in the last 12 months, or both	DHS: 2007
		HHCS: 2008
		MIS: 2006, 2008, 2010, 2012
		Netmark: 2000, 2004
		SBS: 2005, 2009
ITN use or IRS	The proportion of children under 5 years who either slept under an ITN the previous night, or live in a household that was sprayed with an insecticide-based solution in the last 12 months, or both	DHS: 2007
		HHCS: 2008
		MICS: 1999
		MIS: 2006, 2008, 2010, 2012
		Netmark: 2000, 2004
		SBS: 2005, 2009
**Covariates**		
Household electricity	The proportion of households with electricity	Census: 1990, 2000, 2010
		DHS: 1992, 1996–7, 2001–2, 2007
		LCMS: 1996, 1998, 2002–3, 2004–5, 2006, 2010
Female headship of households	The proportion of households with a female head	Census: 1990, 2000, 2010
		DHS: 1992, 1996–7, 2001–2, 2007
		LCMS: 1996, 1998, 2002–3, 2004–5, 2006, 2010
		MICS: 1999
Household size	The average number of members per household	Census: 1990, 2000, 2010
		DHS: 1992, 1996–7, 2001–2, 2007
		LCMS: 1996, 1998, 2002–3, 2004–5, 2006, 2010
		MICS: 1999
Education of women 15 to 44 years old	The average years of schooling for women 15 to 44 years old	Census: 1990, 2000, 2010
		DHS: 1992, 1996–7, 2001–2, 2007
		LCMS: 1996, 1998, 2002–3, 2004–5, 2006, 2010
		MICS: 1999
Improved dwelling wall type	The proportion of households with dwelling walls constructed of an improved material	Census: 1990, 2000, 2010
		DHS: 1992, 1996–7, 2001–2, 2007
		LCMS: 1996, 1998, 2002–3, 2004–5, 2006, 2010
		MICS: 1999
Health facilities per capita	The number of health facilities per 1,000 inhabitants	Census: 1990, 2000, 2010
		JICA: 2006

*Exclusive breastfeeding was selected rather than early initiation or continued breastfeeding because it has the strongest relationship with child mortality.

DHS, Demographic and Health Survey; MIS, Malaria Indicator Survey; MICS, Multiple Indicator Cluster Survey; LCMS, Living Conditions Monitoring Survey; Netmark, Netmark Survey; HHCS, Household Health Coverage Survey; SBS, Sexual Behavior Survey; NMCC, National Malaria Control Centre Administrative Data; Census, National Population Census; JICA, Japan International Cooperation Agency Health Facility Census.

### Data processing

We produced estimates of coverage from each survey-year for each source of data. Our unit of analysis was the district as defined in 2010 for a total of 72 districts. Data collected prior to 2000 referred to a different set of districts totaling 57. For districts that split during the transition from 57 to 72 districts we adjusted estimates from the original district by assuming that the average proportional relationships observed between original and inheriting districts in 2000–2010 applied to the time period 1990–2000 as well. Unlike the earlier Demographic and Health Surveys (DHS) and all three censuses, the 2001–2002 and the 2007 DHS datasets did not contain district identifiers. For the 2007 DHS, the latitude and longitude of each cluster were available and we used these coordinates to identify which district each cluster belonged to. There was no information available in the 2001–2002 survey that allowed us to identify districts, so we used this survey only for province-level estimates.

Except for the Netmark surveys (Table 1), we calculated all indicator estimates according to the definitions in Table 1 using survey microdata, ensuring consistent definitions across sources and taking into account the multistage sampling design for each survey. Surveys provided information for children born up to 5 years before the survey. For ANC, SBA, IPTp immunizations, and EBF, we grouped responses for each child according to year of birth and estimated coverage corresponding to each group for as many years prior to the survey as there were births recorded. Since nationally coordinated programs for ITN distribution and IRS, IPTp, and pentavalent vaccine began in 1999, 2003, and 2005, respectively, we assumed 0.01% coverage for malaria interventions prior to 1997 and for pentavalent immunization prior to 2004. While there were isolated malaria control programs prior to 1997, for example, in Copperbelt province [92], there was no coordinated national effort for malaria control and the vast majority of the population was not covered by ITNs, IRS, or IPTp [93,94].

In addition to survey-based estimates, we calculated IRS coverage from National Malaria Control Centre (NMCC) administrative data by dividing the reported number of structures sprayed by the number of households in the given district-year according to the census. We interpolated the number of households for years between censuses assuming geometric growth.

### Data synthesis

#### Covariates

In many cases, multiple sources for the same year implied different levels for the same covariate. To address this issue and generate a complete time series that synthesized all available data, we used a two-step statistical model. The first step was a linear mixed-effects model which relates the outcome to year and location. The fixed effects of this model included the bases for a natural spline (a method of interpolation using piecewise polynomials) describing the time trend with one interior knot at 2000 while the random effects included a district-level random intercept and random slope. The second step was a Gaussian process regression (GPR) that uses the results from the first stage as the mean function and draws from a multivariate normal distribution, based on the model’s prior and uncertainty in the data, to generate a final estimate for each indicator-district-year. Provincial estimates of indicators were also produced using this method and used as covariates in the first step of the indicator model described below.

### Indicators

A three-step statistical model was used to generate a complete set of indicator estimates, including uncertainty. The first step of the model was an ordinary least squares (OLS) regression of each indicator. Coverage was modeled in logit space to bound the result between 0 and 1. The following model was run separately for each coverage indicator:

$$ logit{(Ind)}_{i,k,t}={\beta}_0+{\beta}_1t+{\beta}_2ele{c}_{i,t}+{\beta}_3 fhea{d}_{i,t}+{\beta}_4 hhsiz{e}_{i,t} + {\beta}_5ed{u}_{i,t} + {\beta}_6wal{l}_{i,t} + {\beta}_7HFP{C}_i + {\beta}_8 In{d}_{k,t}+{\varepsilon}_{i,k,t} $$

where *logit*(*Ind*)_*i*,*k*,*t*_ is the logit-transformed level of coverage for each indicator in district *i*, province *k*, and year *t*; *elec*_*i*,*t*_ is the proportion of households that have electricity in district *i* and year *t*; *fhead*_*i*,*t*_ is the proportion of households with a female head in district *i* and year *t*; *hhsize*_*i*,*t*_ is the mean household size in district *i* and year *t*; *edu*_*i*,*t*_ is the mean years of education for women 15 to 44 years old in district *i* and year *t*; *wall*_*i*,*t*_ is the proportion of dwellings with improved wall type in district *i* and year *t*; *HFPC*_*i*_ is the number of health facilities per capita in district *i*; *Ind*_*k*,*t*_ is the coverage of the indicator at the province level for province *k* and year *t*; and *ε*_*i*,*k*,*t*_ is the error, for district *i*, province *k*, and year *t*.

The second stage in the modeling process involved applying a spatial-temporal regression (ST) to the residuals derived from step 1. ST regression is a form of locally-weighted regression that allows residuals nearby in space and time to have more weight than those farther away. Spatial neighbors were defined as districts within the same province. Temporal neighbors were defined as adjacent data-years within the same district. The predicted residuals from the ST regression are added on to the linear predictions from the OLS regression to generate the mean function used in the final step.

The third and final step is a GPR model with the estimates from the linear and ST regression serving as the mean function. The covariance structure was defined by the Matern Covariance function. We used 1,000 draws from the posterior distribution to calculate estimates of the mean and uncertainty interval (UI). The three step modeling process applied here, including ST and GPR parameters, has been described in detail elsewhere [95] and has been extensively used in global health systematic analyses, most notably in generating many estimates for the Global Burden of Disease study [9,96-98].

We generated estimates at the national level for each indicator by population-weighting the district values. We also estimated an overall measure of composite coverage, based on 10 indicators that reflect the priorities of Zambia’s health system and cover the full range of interventions we studied: the proportion of households with IRS, ITN ownership, or both; IPTp2; EBF; pentavalent, BCG, measles, and polio immunization coverage; ANC4; SBA; and the proportion of children not underweight. Composite coverage can be constructed using theory-based or arbitrary-weighted averages, or latent variable techniques such as factor analysis [48]. For simplicity and ease of interpretation, we chose to apply equal weights to all interventions, and constructed composite coverage as the simple average of the 10 interventions. We also explored the relationship between socioeconomic status, measured as a composite of four socio-demographic variables^b^, and composite coverage, and report the Pearson correlation between these measures across districts and years. In the results section, we present findings for the 12 indicators that are priorities in the Zambian health system. Results for the additional 5 indicators estimated in this analysis are presented in Additional files 2 and 3.

### Ethical approval

Permission to implement this research project was obtained from the Ministry of Health of Zambia. Ethical approval for this study was obtained from the institutional review board of the University of Washington. The study was conducted in compliance with national regulatory and ethics guidelines.

## Results

### Individual interventions

**Figure 1:**
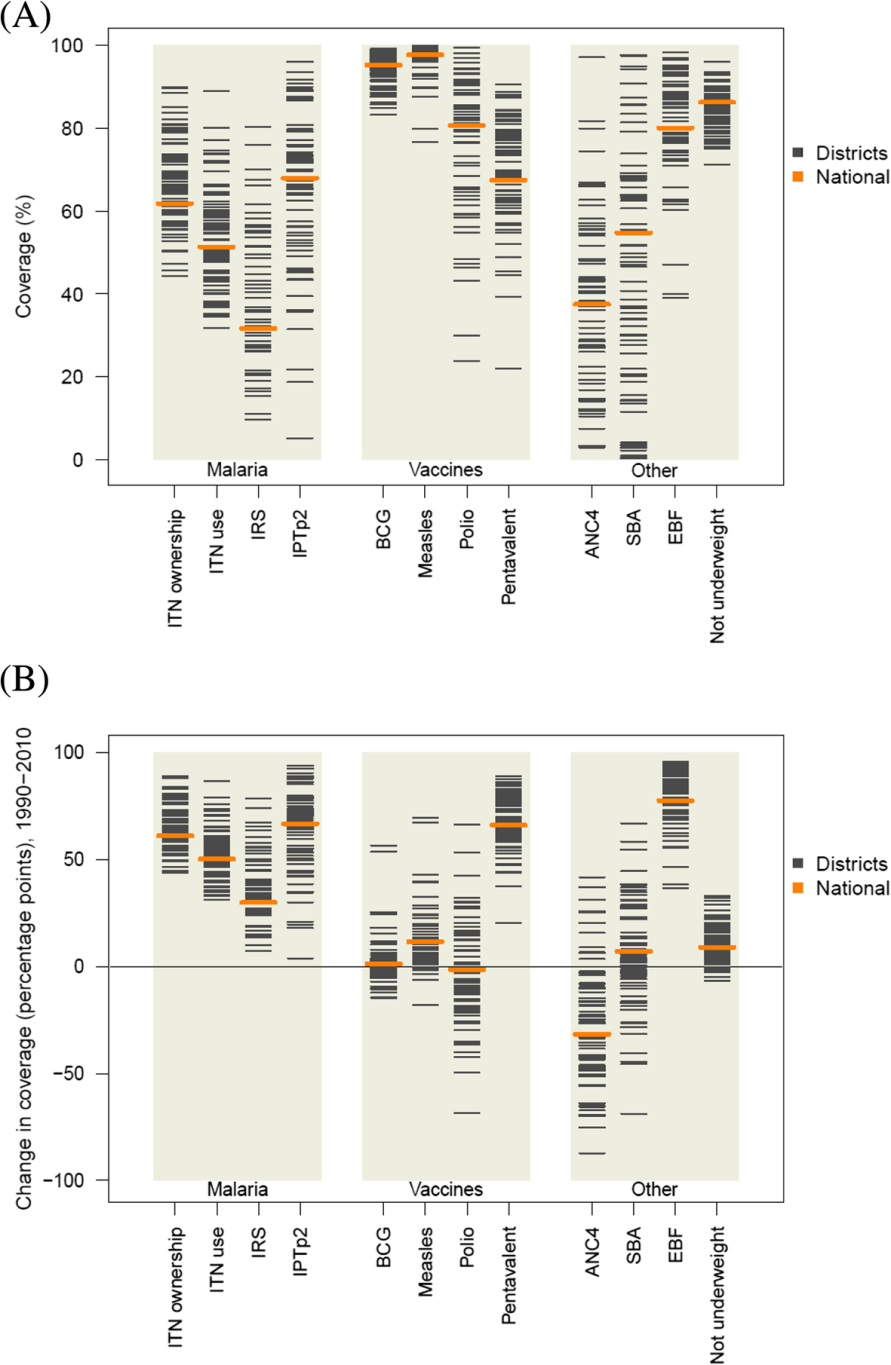
**Distribution of intervention coverage in 2010 (A) and absolute change in coverage from 1990 to 2010 among districts (B).** IRS displayed only for targeted districts in 2010.

**Figure 2:**
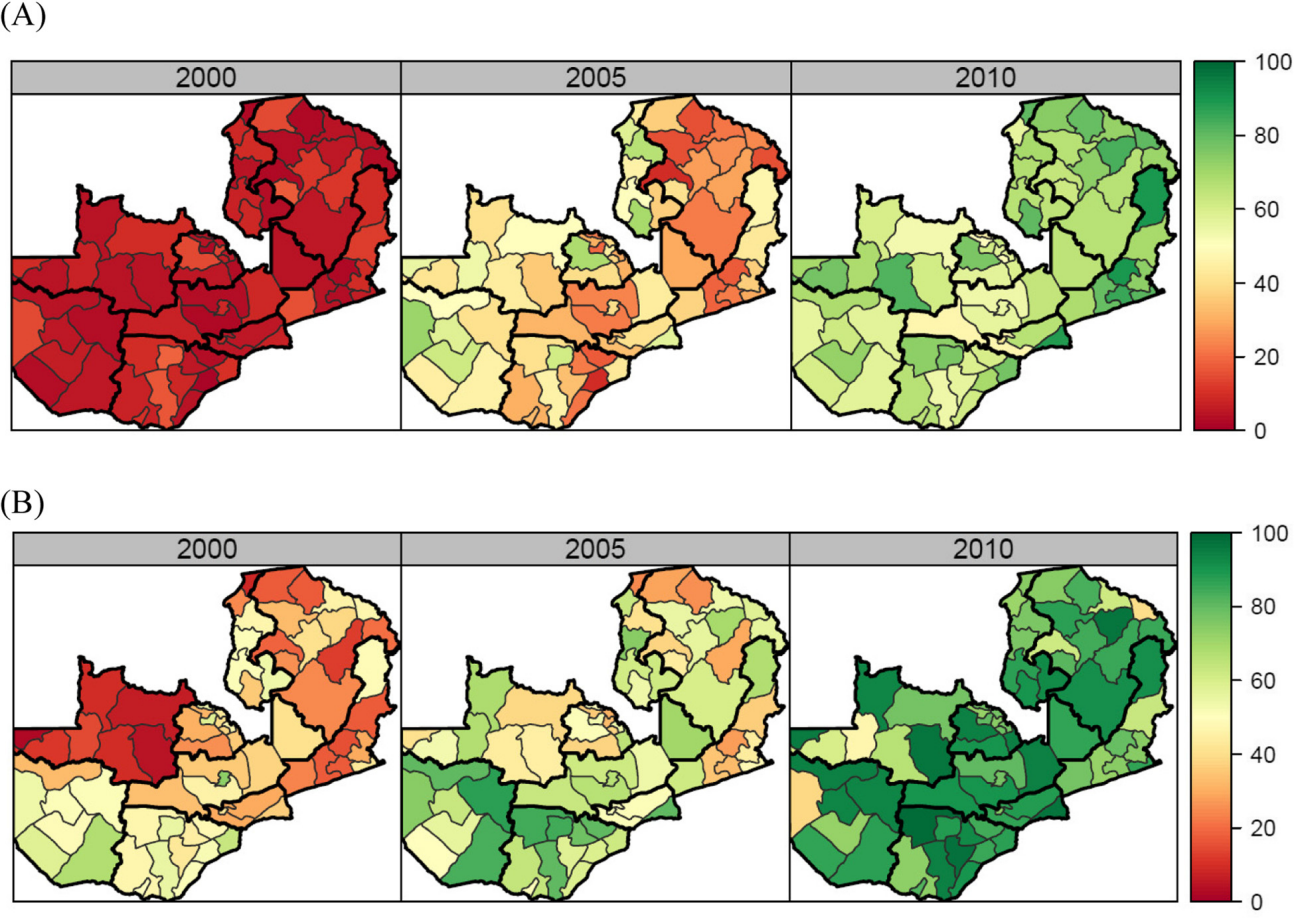
**Coverage of insecticide-treated net ownership (A) and exclusive breastfeeding (B) by district in 2000, 2005, and 2010.**

We found a wide variation in both the levels of coverage and average change between 1990 and 2010 across the 12 indicators shown in Figure 1. For malaria control interventions, the scale-up was remarkable across Zambia (Figure 2A). At the same time, in 2010 ITN ownership ranged from 44% (95% UI: 42–47%) to 90% (95% UI: 85–93%), while use of ITNs by children under the age of 5 years exhibited an even larger range (from 32% [95% UI: 25–40%] to 89% [95% UI: 81–94%]). IPTp2 levels rose rapidly in many districts, but leveled off or experienced declines in coverage in some districts after 2007, and as a result coverage levels ranged from 5% (95% UI: 2–11%) to 96% (95% UI: 92–98%) in 2010.

For immunizations, Zambia maintained high levels of BCG and measles immunization coverage across districts, but polio immunization coverage was highly variable across districts, with a range in 2010 from 24% (95% UI: 11–42%) in Mufumbwe to 99% (95% UI: 98–100%) in Chavuma, both in North-Western province. While at the national level coverage did not change significantly between 1990 and 2010, remaining around 81%, more than half of districts in Zambia had lower levels of polio immunization in 2010 compared to 1990 (Figure 1B). We found similarly large disparities in pentavalent vaccine coverage across districts in 2010; as the vaccine was nationally launched in 2005, this finding likely reflects differential uptake of a new intervention throughout Zambia.

For the other MCH interventions included in this analysis, particularly notable progress was made for EBF (Figure 2B). On average across districts, EBF increased by 79 percentage points, with significant progress observed across all districts (Figure 1B). Districts in Southern province experienced the largest improvements, showing an average 85 percentage point increase in EBF between 1990 and 2010. Progress was also made on malnutrition: the proportion of children who are underweight decreased during this time period and the range across districts is smaller than for any other indicator included in this analysis.

**Figure 3:**
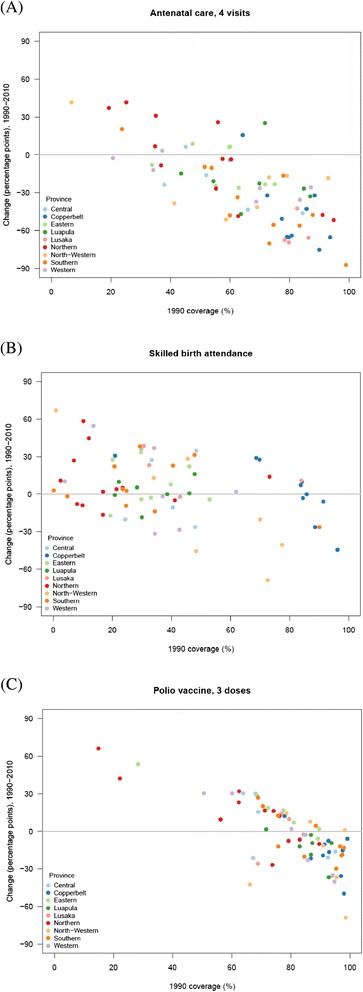
**Absolute change in coverage between 1990 and 2010 compared with estimated coverage in 1990 for (A) antenatal care, 4 visits, (B) skilled birth attendance, and (C) polio immunization, by district.** Each dot represents a district.

On the other hand, ANC4 and SBA displayed the largest differences in levels and trends. In 2010, the difference between the highest-performing and lowest-performing districts was 86 percentage points for ANC4 and 97 percentage points for SBA. At the same time, national ANC4 decreased by 31 percentage points between 1990 and 2010, and declining trends in many districts were also observed for SBA and polio immunization coverage, shown in more detail in Figure 3. During this 20-year period, 59 (of 72) districts in Zambia experienced declines in ANC4, 31 experienced declines in SBA, and 41 experienced declines in polio immunization. The number of districts with declining coverage is particularly worrisome for polio immunization since coverage dropped in several districts considered at high risk for polio importation from neighboring countries [99]. Figure 3 also highlights that the highest-performing districts in 1990 tended to have the largest declines over the next two decades, while districts with lower baseline coverage achieved the greatest gains. The correlation coefficients between coverage level in 1990 and change between 1990 and 2010 were −0.73 for ANC4, −0.37 for SBA, and −0.76 for polio immunization. Geographic patterns in indicator trends are also notable: districts in Copperbelt province (show in dark blue) had relatively high baseline levels and large declines, while districts in Northern province (dark red) largely had lower baseline levels in 1990 but experienced gains.

### Composite coverage

**Figure 4:**
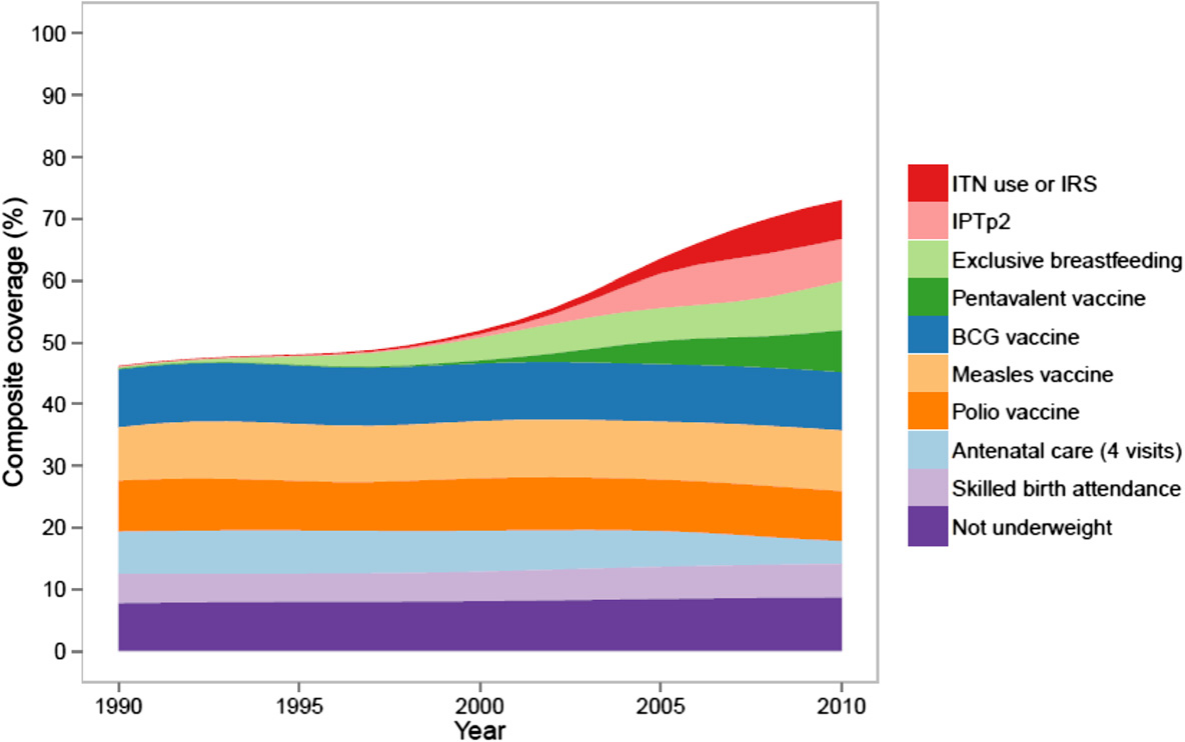
**National composite coverage by intervention composition, 1990 to 2010.**

Figure 4 shows the national trend in composite coverage by its component interventions. If coverage of all 10 interventions measured here was 100%, then composite coverage would be at 100%. While overall composite coverage increased from 46% in 1990 to 73% in 2010, with more substantial gains in the early 2000s, most of the expansion in composite coverage is due to the scale-up of malaria control, EBF, and pentavalent immunization. Polio immunization, ANC4, and SBA exhibited minimal progress and in some cases declined.

**Figure 5:**
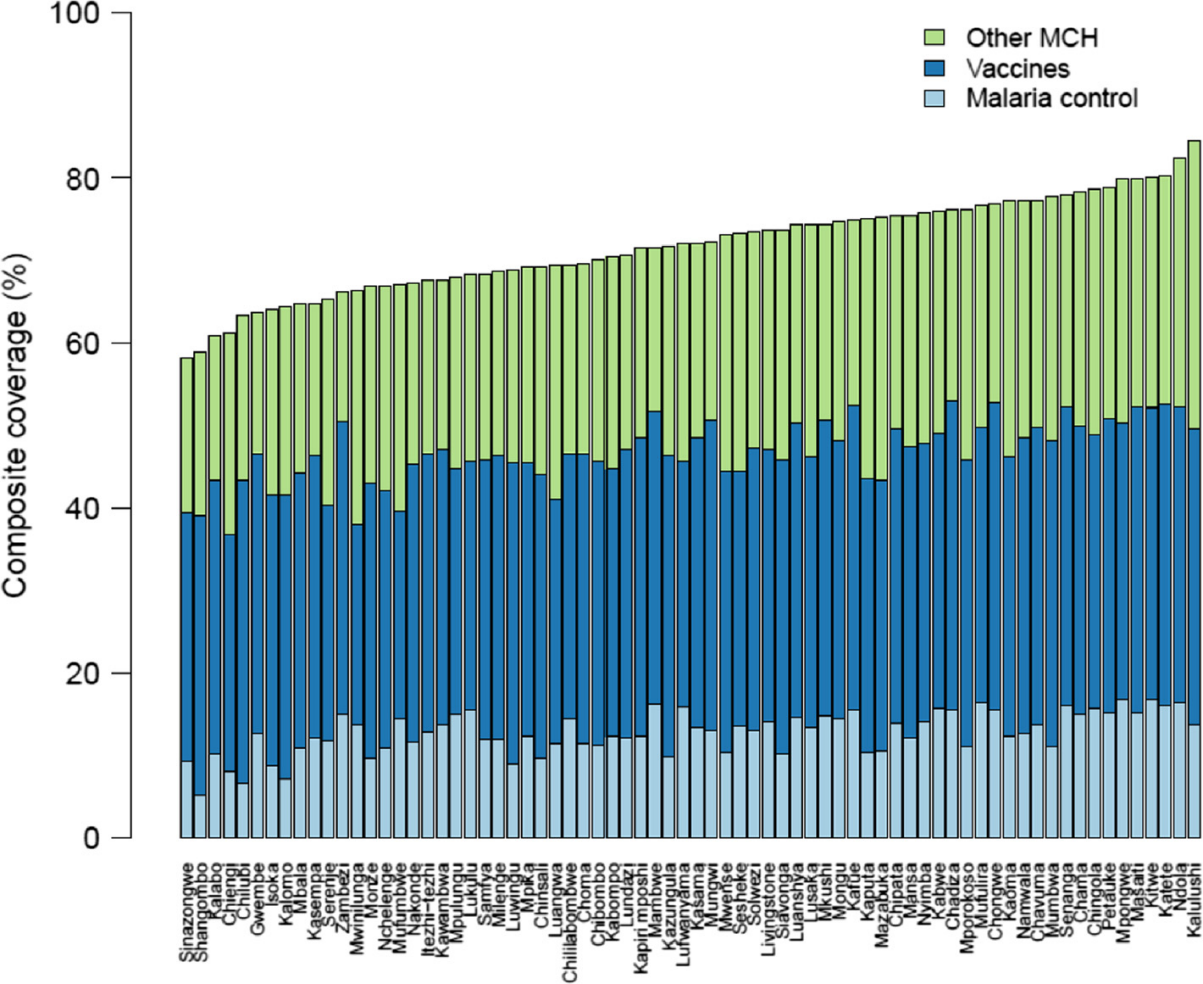
**Composite coverage by district and intervention cluster, 2010.** Other MCH: ANC4, SBA, EBF, and proportion of children not underweight. Vaccines: BCG, measles, polio, and pentavalent. Malaria control: ITN ownership or IRS and IPTp2.

This national trend also masked substantial heterogeneity across districts. While the absolute difference in composite coverage between the highest-performing and lowest-performing districts declined from 37 to 26 percentage points between 1990 and 2010, considerable variation in composite coverage persisted across districts in 2010 (Figure 5). Composite coverage ranged from 58% in Sinazongwe to 85% in Kalulushi; nevertheless, Figure 5 shows that the relative contributions of malaria control, immunizations, and other MCH interventions were actually quite similar across districts. Surprisingly, the bivariate correlation between composite coverage and socioeconomic status was only 0.43.

## Discussion

The findings from this first-ever assessment of levels and trends in coverage of key MCH interventions across districts in Zambia show that substantial progress has been made in scaling up interventions such as malaria control and pentavalent immunization, as well as EBF. At the same time, stagnation and declines of intervention coverage occurred for routine services such as ANC4, SBA, and polio immunization. This district-level analysis revealed marked inequalities in coverage, particularly for maternal health interventions. Rates of progress for routine services varied substantially across districts, while rapidly scaled up interventions showed more uniform improvements across the country. Benchmarking performance of districts in delivering key interventions offers important insights and action points for policymakers, enabling them to identify underperforming districts and interventions with declining trends, as well as understand the largest disparities across districts.

This study revealed that very few districts in Zambia were high performers for all intervention types and, in general, performance was not highly correlated with average district socioeconomic status. While a few districts stand out as having uniformly low coverage and require immediate attention (Chiengi, Samfya, Sinazongwe, and Shang’ombo), several districts had mixed success. For example, coverage in Lusaka (the country’s capital district, a region with lower malaria transmission intensity) in 2010 was above the national average for IRS, IPTp2, BCG, measles, and polio immunization coverage, and SBA, but it was well below the national average for ITN ownership and use, pentavalent immunization, ANC4, and EBF. In contrast to other countries where similar studies have been undertaken, geographic patterns in coverage did not reflect geographic variation in socioeconomic status. For example, in the United States [100-102], Mexico [48], and China [50], patterns in health indicators largely mirror geographic variation in socioeconomic status and tend to be uniform across indicators; that is, wealthier regions within each country tend to have high life expectancy and high coverage of interventions such as in-facility delivery and hypertension treatment. In the case of Zambia, the fact that we did not see a strong association between socioeconomic status and coverage suggests that more complex factors are at work. Further investigation is needed to understand the drivers of the variation in performance across districts. In particular, more detailed case studies of districts with heterogeneous performance are needed to elucidate the reasons why certain interventions but not others are successful in these contexts. Zambia’s experience highlights the importance of benchmarking to identify regions of high and low performance for a variety of key health indicators.

This work highlights two major patterns in health system performance across districts in Zambia: success in scaling up vertical programs and stagnation, or even weakening, of horizontal programs. At the national and district level, Zambia achieved greater successes in newer, rapidly scaled up interventions while gains in routine services delivery either stalled or declined, raising concerns that successes in vertical programs may have come at the expense of primary health care. Other key interventions such as HIV/AIDS treatment, prevention of mother-to-child transmission of HIV, and case management of childhood malaria are not covered in the present study due to data limitations, but were also scaled up dramatically in this time period, and may have also contributed to our observed trends in routine service provision. Although there is some evidence that such a ‘crowding-out effect’ did not occur [103,104], the stark contrast between intervention coverage trends from horizontal and vertical programs warrants further examination. Concerns about vertical programs displacing horizontal ones are not unique to Zambia. A key challenge as LMIC health systems grow and the emphasis on UHC is heightened is to balance the roles of vertical and horizontal programs and ultimately leverage both to strengthen overall health system performance in each country. The Mexican health system reform is a notable example of success for implementing a more ‘diagonal’ approach, employing cost-effective interventions that link health facilities to community health needs, and benefiting from a balance between strong primary health care and vertical programs [105]. Zambia, along with many other countries, has begun to make the shift to diagonal programs that combine the strengths of disease-specific and comprehensive delivery systems [106].

This study underscores the importance of incorporating equity goals in target-setting. Zambia’s scale-up of malaria control at the national level is impressive, but its emphasis on equity is lacking. The country’s *National Malaria Strategic Plan 2006–2010* set several malaria intervention coverage targets for the country to achieve by 2010 [83]. These targets were very ambitious, and despite marked progress since 2000, no district achieved all four targets in 2010. Zambia, as well as other countries, would greatly benefit from formulating health policy goals with explicit mention of targets for each district or region, as this would ensure that particular attention and resources are directed to the poorest performing regions. Zambia’s *National Health Strategic Plan 2011–2015* [82] emphasizes UHC, equity, and overall health system strengthening, but does not incorporate specific subnational targets. Achievement of the plan’s targets (e.g., to reduce the national under-5 mortality rate from 119 deaths per 1,000 live births to 63 deaths per 1,000 live births by 2015) will require targeting interventions to the most disadvantaged populations. With the appropriate framing and implementation, this plan could be used as a platform to promote greater within-country equality. At the global level, it is well recognized that the lack of clearly incorporating equity into the Millennium Development Goals (MDGs) was a major oversight, one that should not be repeated in the finalization of the forthcoming Sustainable Development Goals (SDGs) [107-109]. The experience of the MDGs warns that national target-setting not only fails to represent those most in need, but that such exercises can actually incentivize the opposite, to target the most accessible populations and to potentially propagate even greater inequalities [110-112].

Incorporating equity into global and national targets has significant implications for data collection systems. Data quality and availability limited the scope of this study, and a future global focus on subnational benchmarking will require substantial strengthening of existing and emerging data collection systems. In this analysis, the generation of district trends in coverage was challenging, and required triangulating information from many sources and applying sophisticated statistical techniques. Zambia is a comparatively data-rich country within sub-Saharan Africa, but most countries, both developed and developing, are not well-equipped for the routine collection and monitoring of data at the most relevant administrative levels. The demand from governments, international agencies, donors, civil society groups, and the public for high quality health information is growing rapidly and existing data collection systems are not keeping pace [113,114]. The MDGs motivated significant improvements in country-level monitoring of key health indicators, but the overarching evidence base and state of data collection systems, particularly in developing countries, remains weak. If the SDGs ultimately include subnational targets, a similar data revolution will be necessary. In order to report on subnational targets for a variety of indicators, data collection systems will need to become more integrated, cover a finer array of geographic regions and health topics, include measures of quality of interventions (such as biomarkers and health examinations), and encourage regular validation and use of the information collected for policymaking.

### Limitations

The findings of this study need to be interpreted within the context of the limitations we encountered. First, the coverage of several key interventions could not be estimated in this analysis due to lack of data. Case management of childhood diarrhea, pneumonia, and malaria could not be estimated because caregivers reported too few cases per survey-district-year. Second, data on additional indicators from several administrative sources (i.e., the NMCC’s ITN distribution database; National AIDS Council quarterly service reports; and Medical Stores Limited drug supply database) were excluded due to concerns about accuracy, completeness, and lack of appropriate denominator data. Third, our estimates of intervention coverage do not reflect the quality of the intervention received or any health gains associated with receiving the intervention. This a critical input for determining the effectiveness of health service provision and understanding whether the receipt or use of an intervention translates into improved health outcomes. Fourth, the findings from this study are largely based on self-reported information from household survey respondents, and thus are prone to biases related to self-reported data. Fifth, we encountered small sample sizes for some indicators from surveys that were not designed to be representative at the district level; for these indicators, coverage estimates were generally accompanied by larger levels of uncertainty. Finally, the analysis presented here did not seek to evaluate the causes of declines, improvements, or differences in coverage across districts and over time. Understanding the drivers of these trends in intervention coverage is critical, and it is likely that much could be learned by conducting a rigorous assessment of these changes.

## Conclusions

Subnational benchmarking is important for assessing progress towards UHC, identifying drivers of success, and prioritizing areas of greatest need. This study shows that Zambia saw notable gains in the delivery of malaria control interventions, BCG and measles immunization, and EBF across districts, with small differences in the levels of coverage achieved. On the other hand, for SBA, ANC4, and polio and pentavalent immunization, the gap between the highest-performing and lowest-performing districts was very large. Geographic patterns in intervention coverage were not highly correlated with socioeconomic status, and further investigation is needed to understand what is driving such heterogeneity at the district level. Subnational analyses, such as the work presented here, should be conducted regularly so that the findings they generate can directly inform policy decisions and increase accountability at all levels of the health system and government.

## Endnotes

^a^Stunting reflects chronic under-nutrition and wasting reflects acute under-nutrition. We selected underweight because it is representative of both chronic and acute under-nutrition and is the preferred World Health Organization measure of malnutrition [86].

^b^These variables include mean years of education among adults aged 18 and older, coverage of improved sanitation, coverage of improved cooking fuel, and household electricity availability.

## Additional files

Additional file 1:
**is a table of data sources.**
Additional file 2:
**is an excel file of intervention coverage and outcome estimates for all districts from 1990 to 2010.**
Additional file 3:
**features graphics accompanied by a scale that represents intervention coverage or the proportion of children who were underweight ranging from 0% to 100%.**

## Abbreviations

ANC: Antenatal care
BCG: Bacillus Calmette-Guérin
DHS: Demographic and health survey
DPT: Diphtheria-pertussis-tetanus
EBF: Exclusive breastfeeding
GPR: Gaussian process regression
IPTp: Intermittent preventive therapy for malaria during pregnancy
IRS: Indoor residual spraying
ITN: Insecticide-treated net
LMIC: Low- and middle-income countries
MDGs: Millennium Development Goals
MCH: Maternal and child health
NMCC: National Malaria Control Centre
OLS: Ordinary least squares
SBA: Skilled birth attendance
SDGs: Sustainable Development Goals
ST: Spatial-temporal
UHC: Universal Health Coverage
UI: Uncertainty interval
