# Supplementary material for: Benchmarking health system performance across districts in Zambia: a systematic analysis of levels and trends in key maternal and child health interventions from 1990 to 2010
Source: BMC Med. 2015 Apr 2;13:69. doi: 10.1186/s12916-015-0308-5 (PMC4382853; doi:10.1186/s12916-015-0308-5)
Supplement: Additional file 1: — is a table of data sources. [file 12916_2015_308_MOESM1_ESM.docx]

**Supplemental Figures**

**Figure S1: Coverage for all indicators, 1990, 2000, and 2010**

**Antenatal care (1 visit)**

**
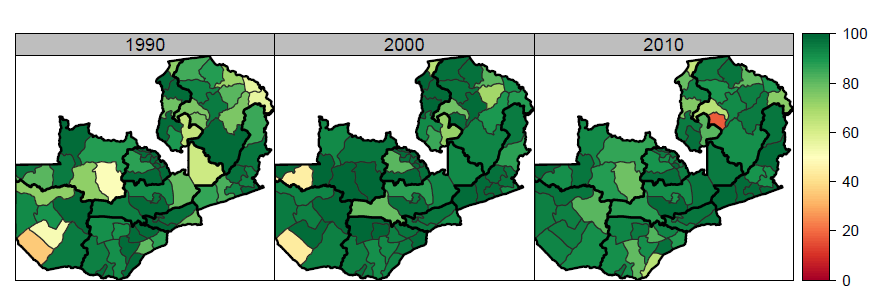
**

**Antenatal care (4 visits)**

**
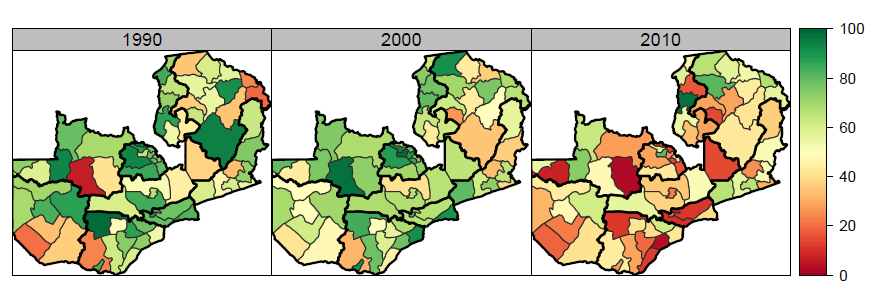
**

**BCG immunization (1 dose)**

**
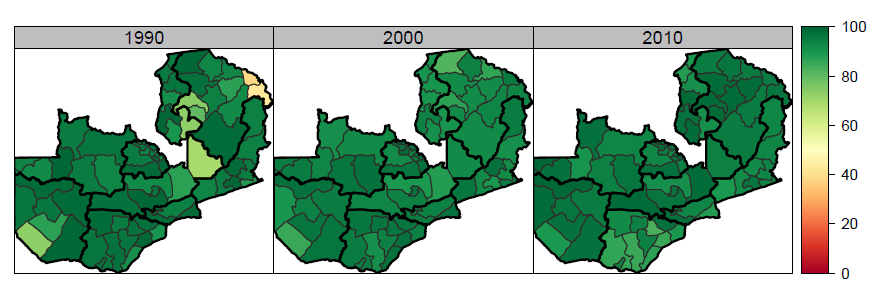
**

**DPT immunization (3 doses)**

**
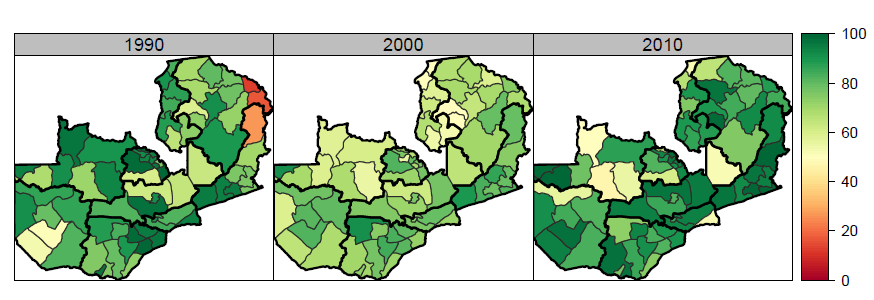
**

**Exclusive breastfeeding**

**
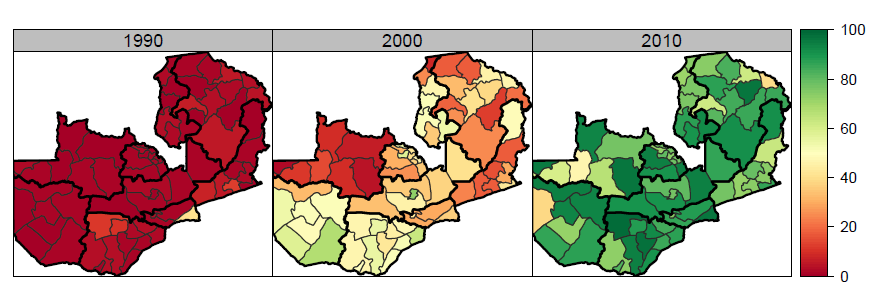
**

**Intermittent preventive therapy for malaria during pregnancy (1 dose)**

**
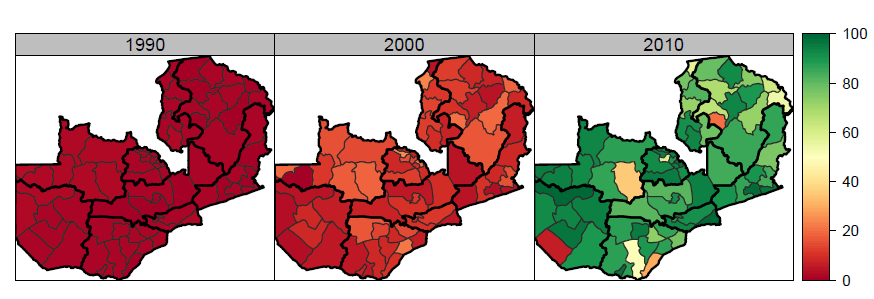
**

**Intermittent preventive therapy for malaria during pregnancy (2 doses)**

**
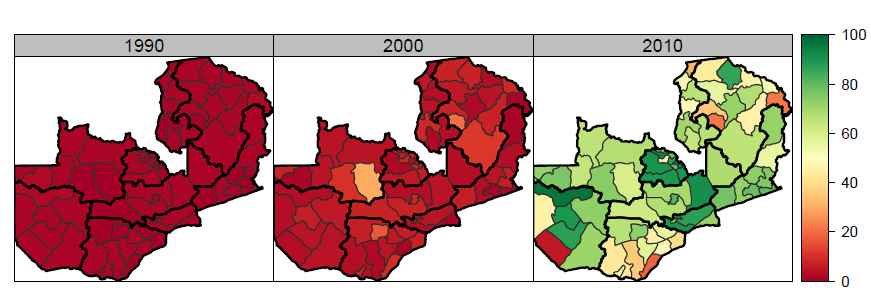
**

**Indoor residual spraying**

**
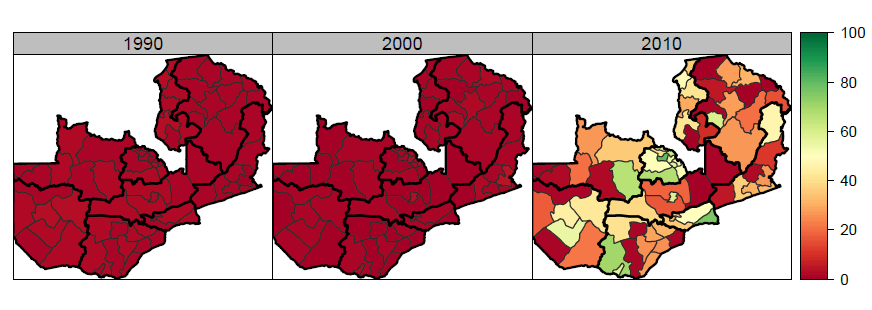
**

**Insecticide-treated net ownership**

**
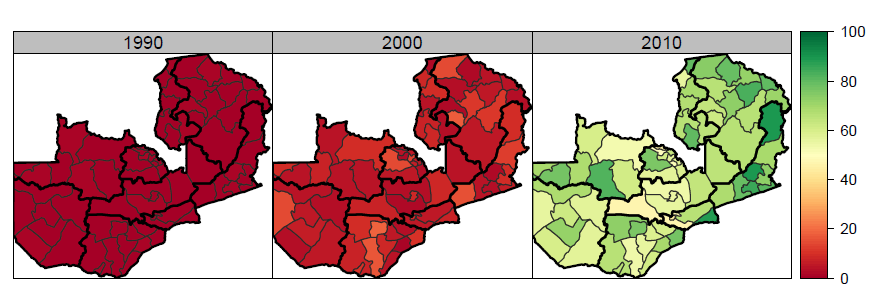
**

**Insecticide-treated net ownership or indoor residual spraying**

**
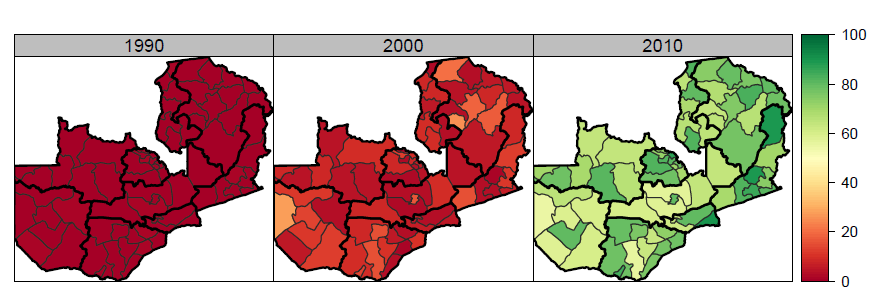
**

**Insecticide-treated net use**

**
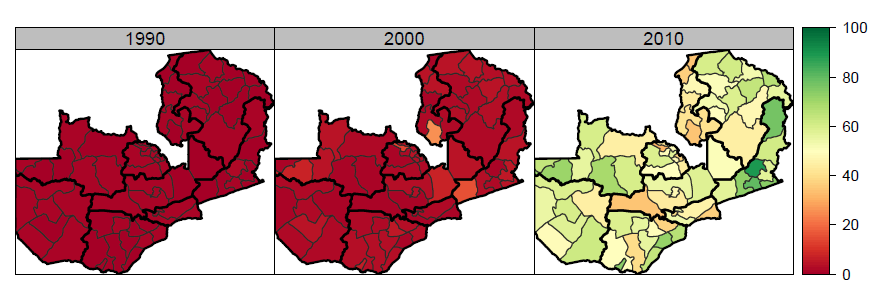
**

**Insecticide-treated net use or indoor residual spraying**

**
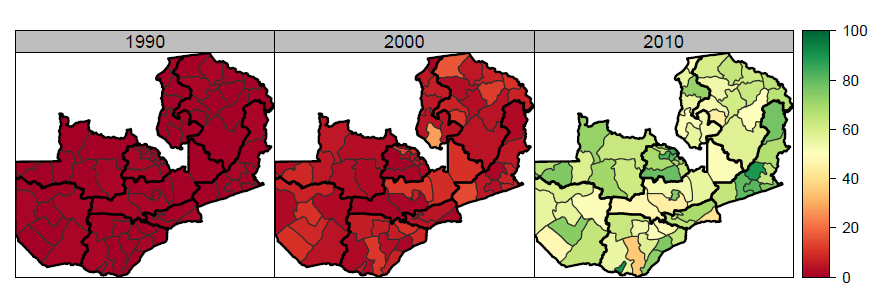
**

**Measles immunization (1 dose)**

**
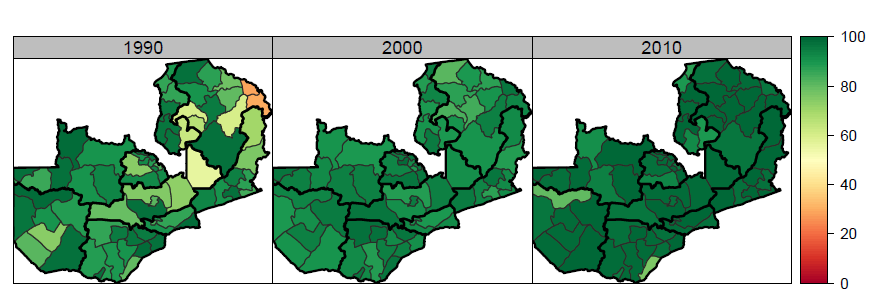
**

**Pentavalent immunization (3 doses)**

**
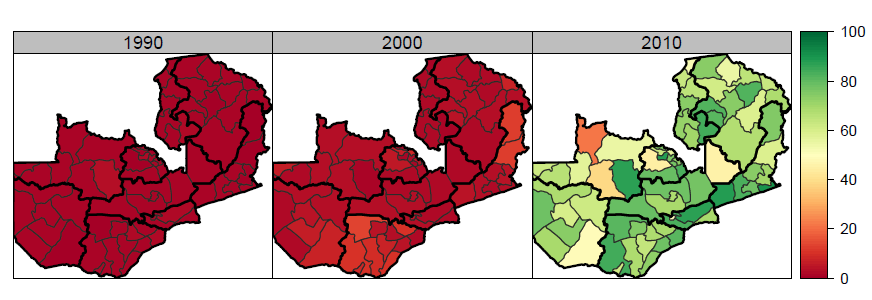
**

**Polio immunization (3 doses)**

**
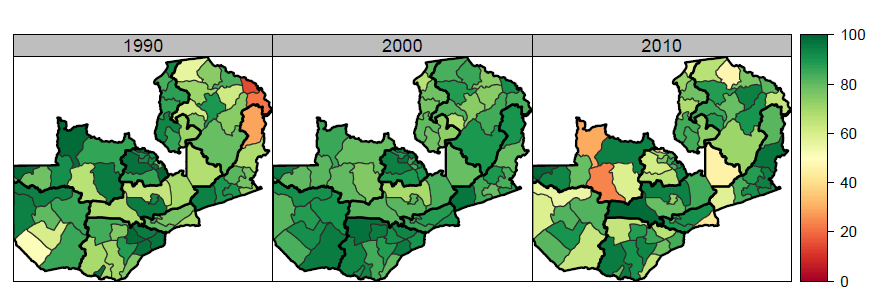
**

**Skilled birth attendance**

**
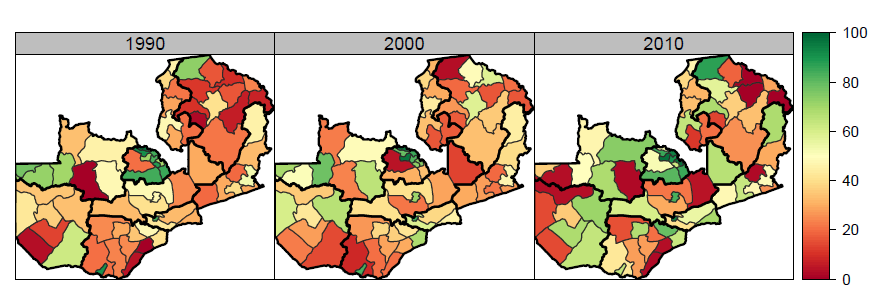
**

**Underweight**

**
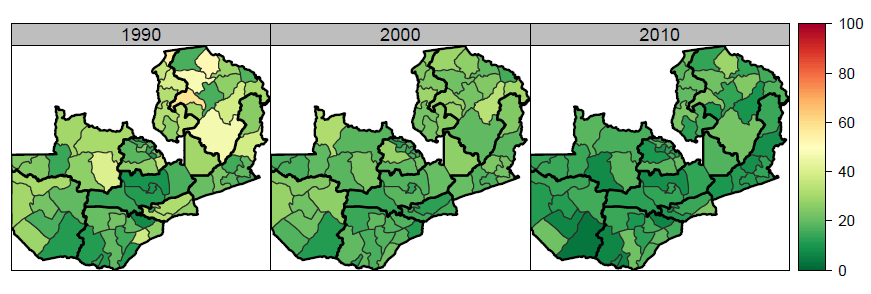
**

**Figure S2: Coverage for all indicators, 2000, 2005, and 2010**

**Antenatal care (1 visit)**

**
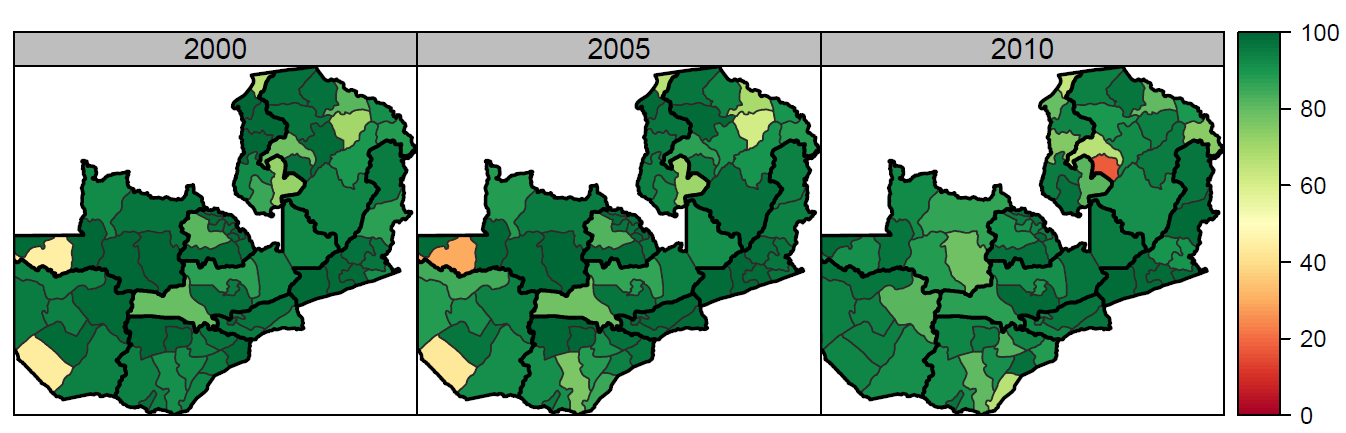
**

**Antenatal care (4 visits)**

**
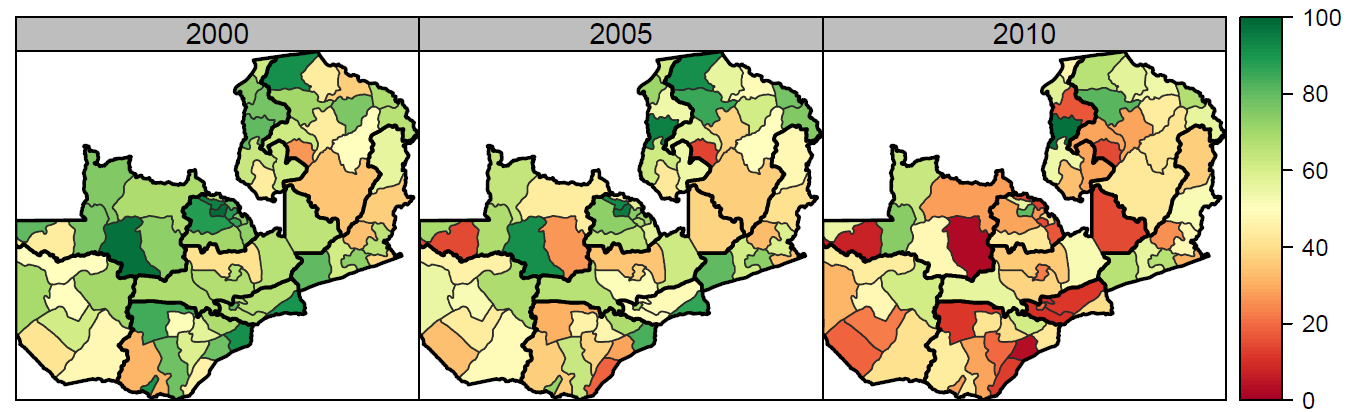
**

**BCG immunization (1 dose)**

**
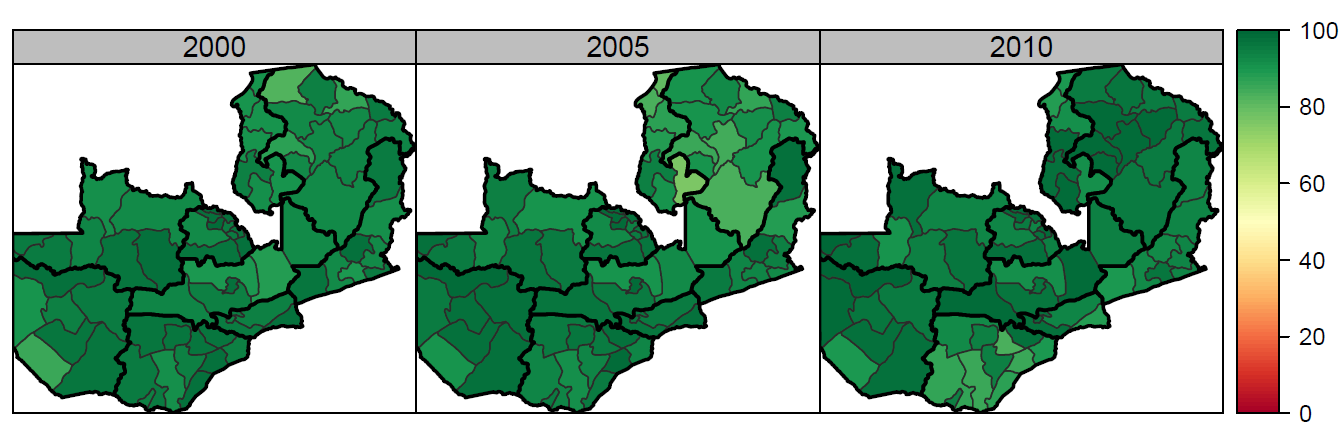
**

**DPT immunization (3 doses)**

**
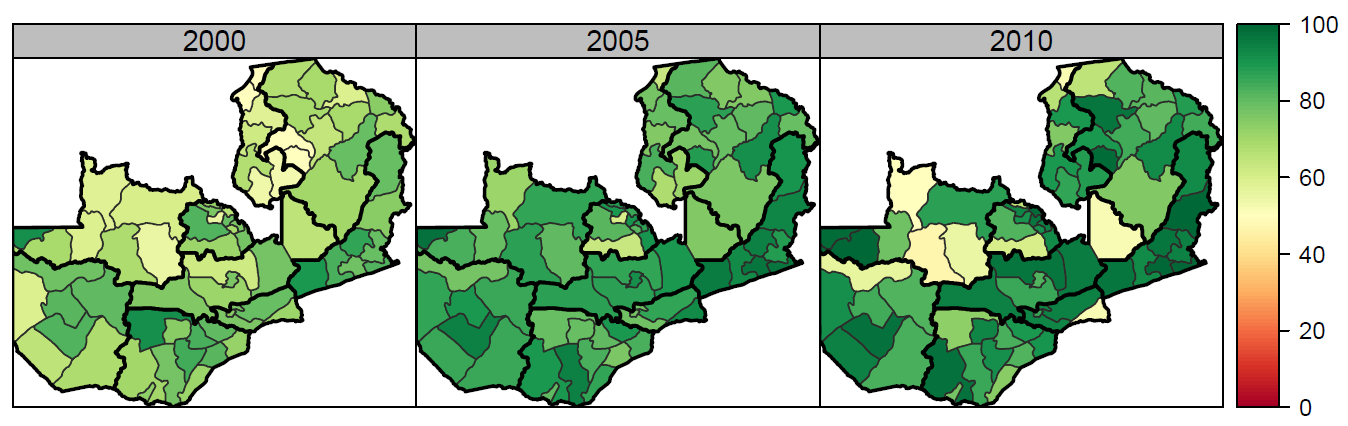
**

**Exclusive breastfeeding**

**
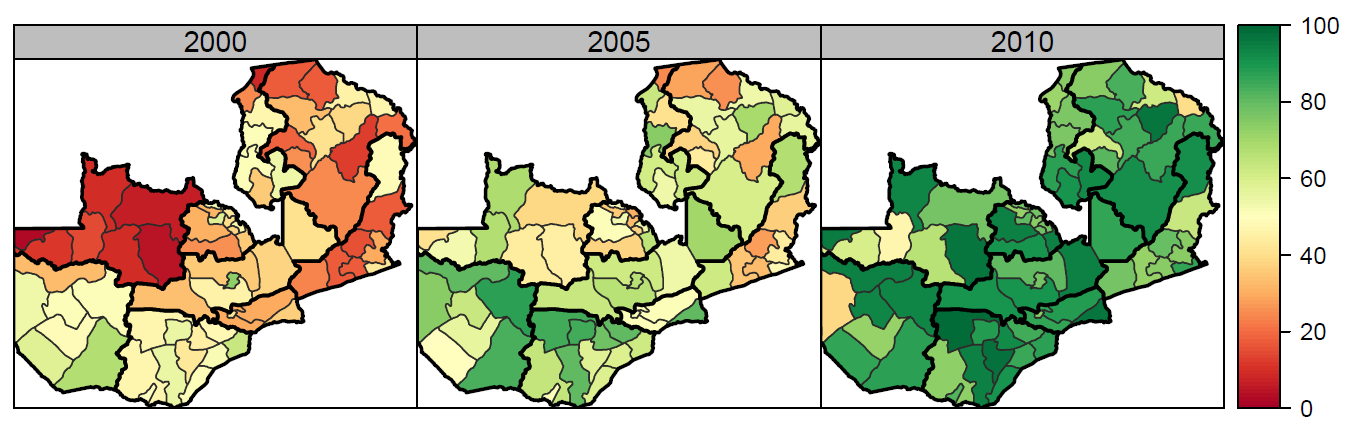
**

**Intermittent preventive therapy for malaria during pregnancy (1 dose)**

**
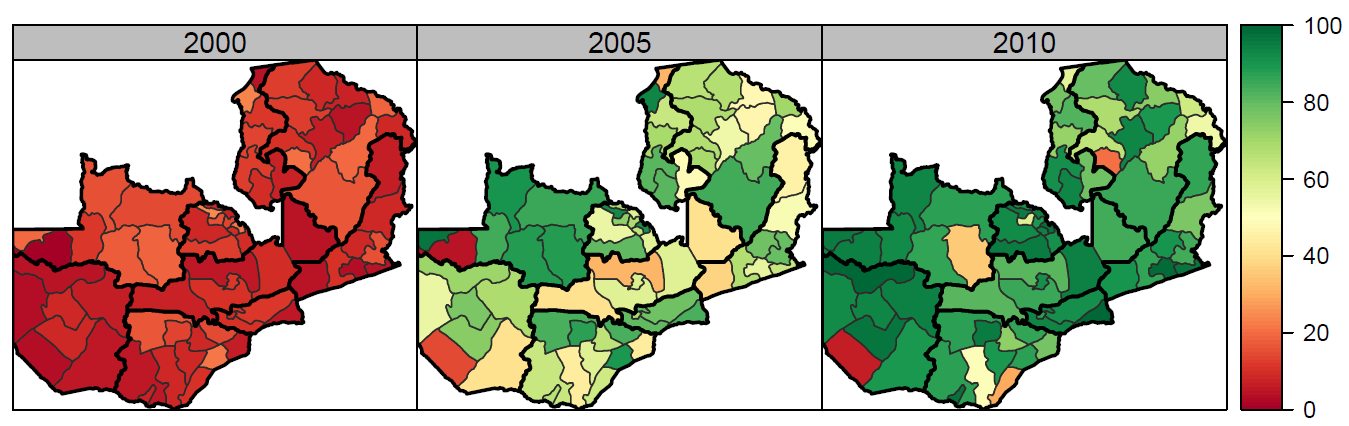
**

**Intermittent preventive therapy for malaria during pregnancy (2 doses)**

**
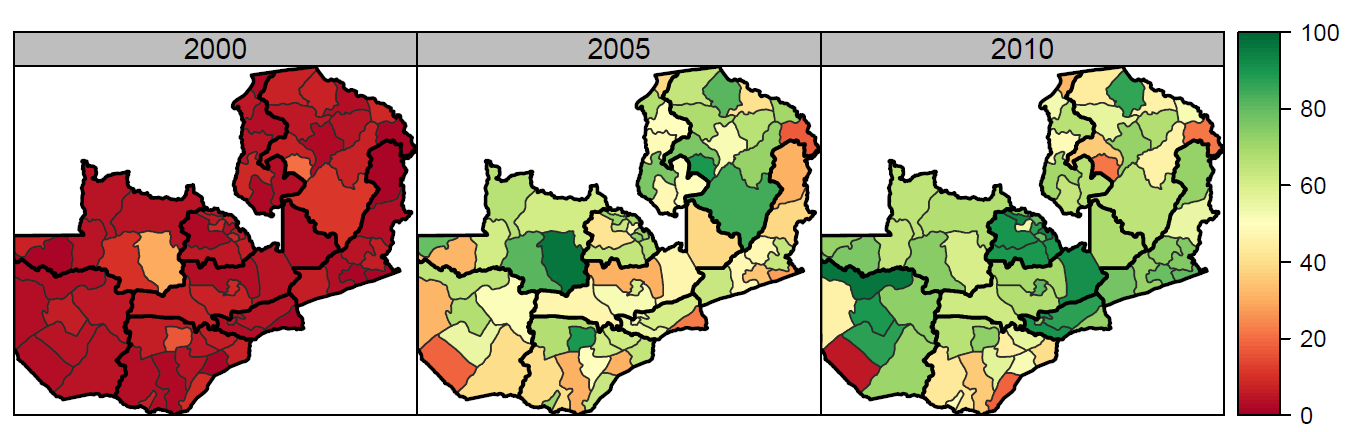
**

**Indoor residual spraying**

**
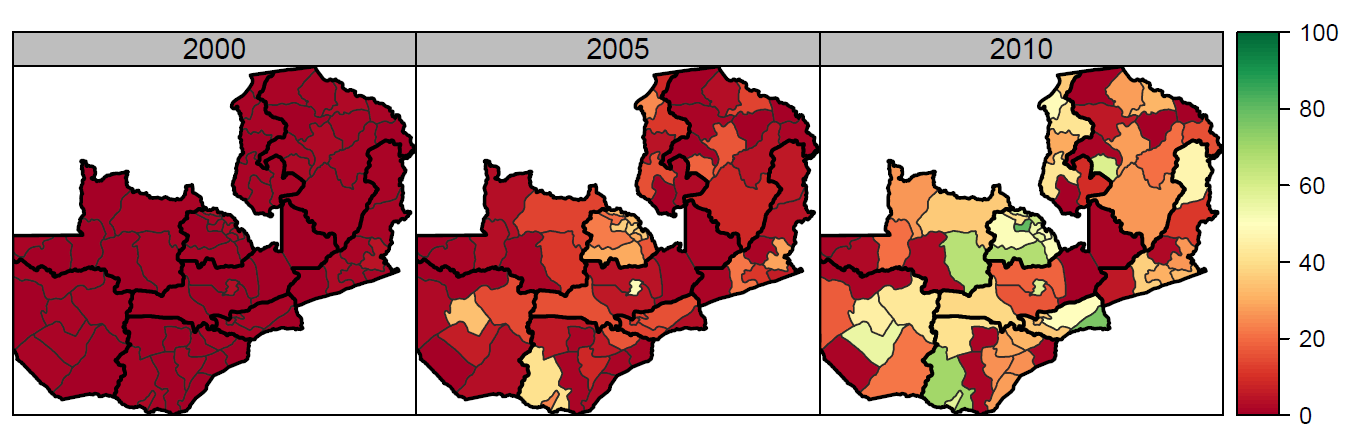
**

**Insecticide-treated net ownership**

**
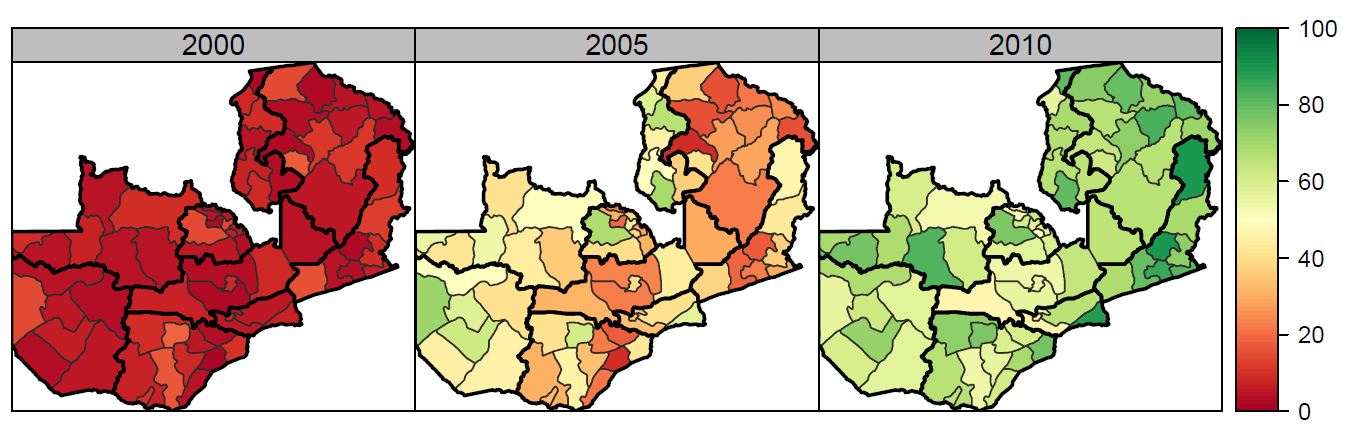
**

**Insecticide-treated net ownership or indoor residual spraying**

**
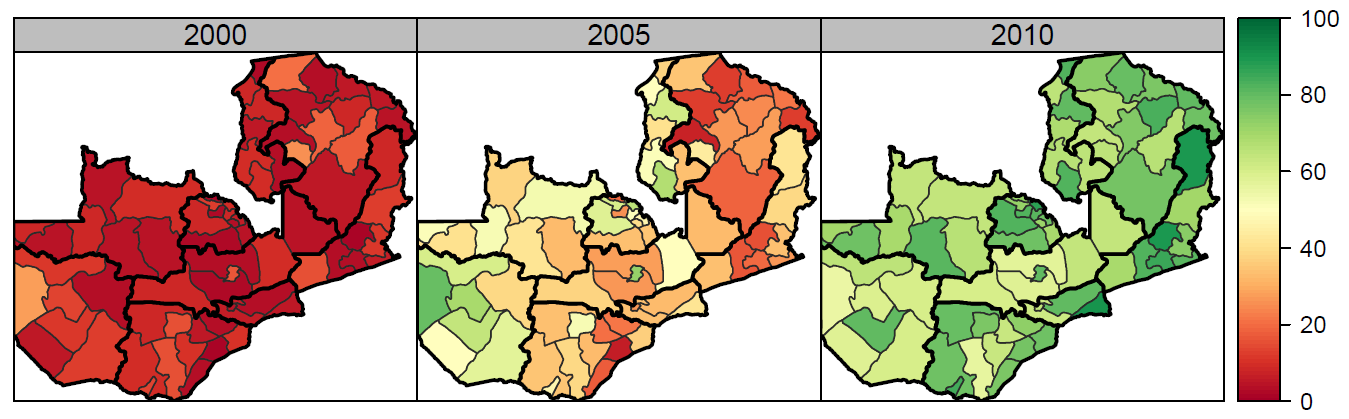
**

**Insecticide-treated net use**

**
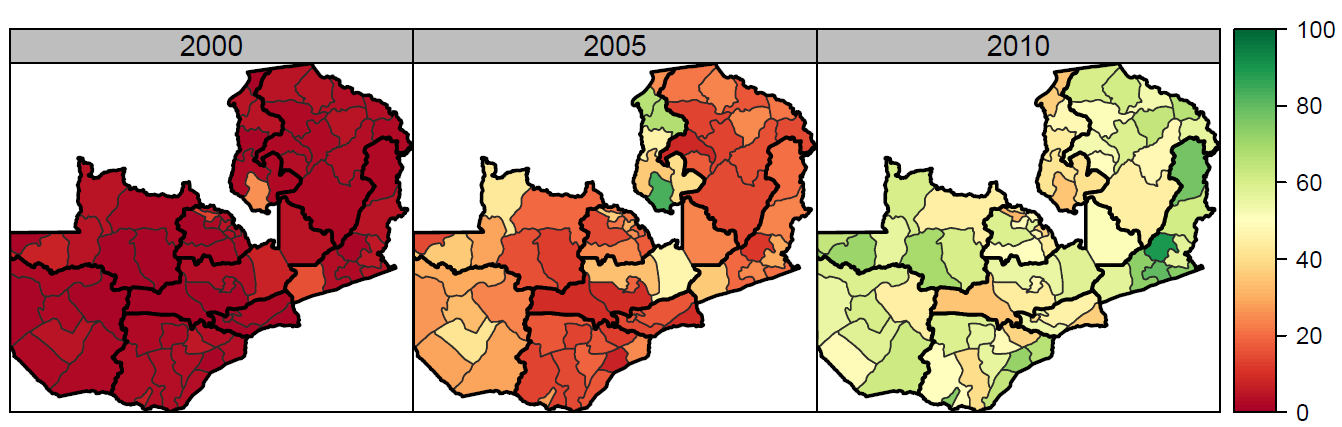
**

**Insecticide-treated net use or indoor residual spraying**

**
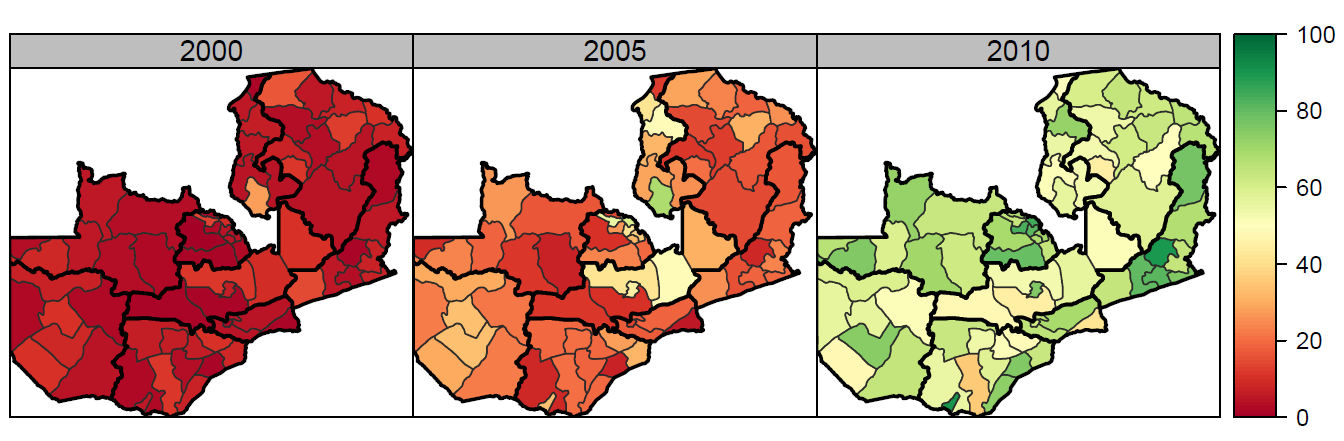
**

**Measles immunization (1 dose)**

**
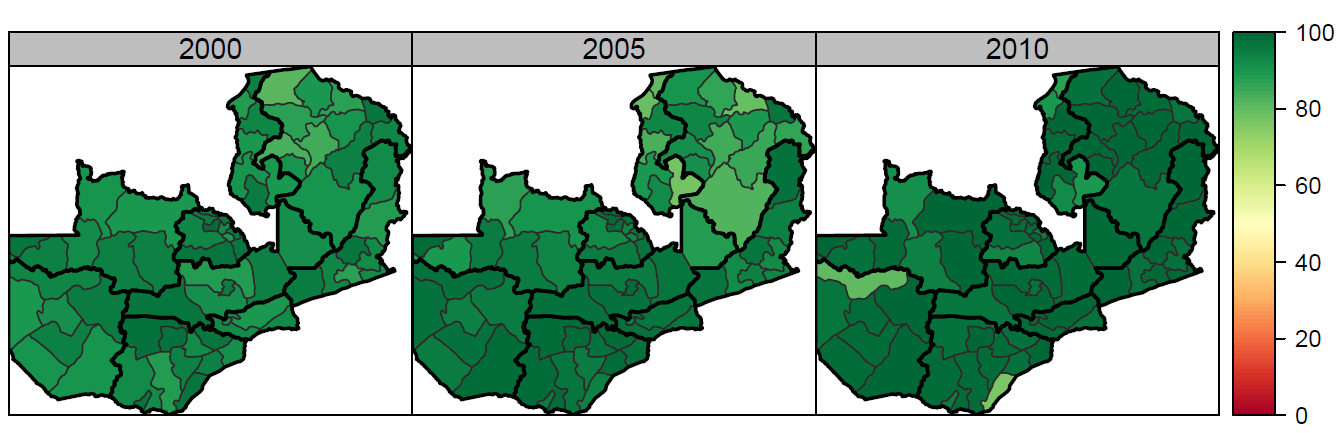
**

**Pentavalent immunization (3 doses)**

**
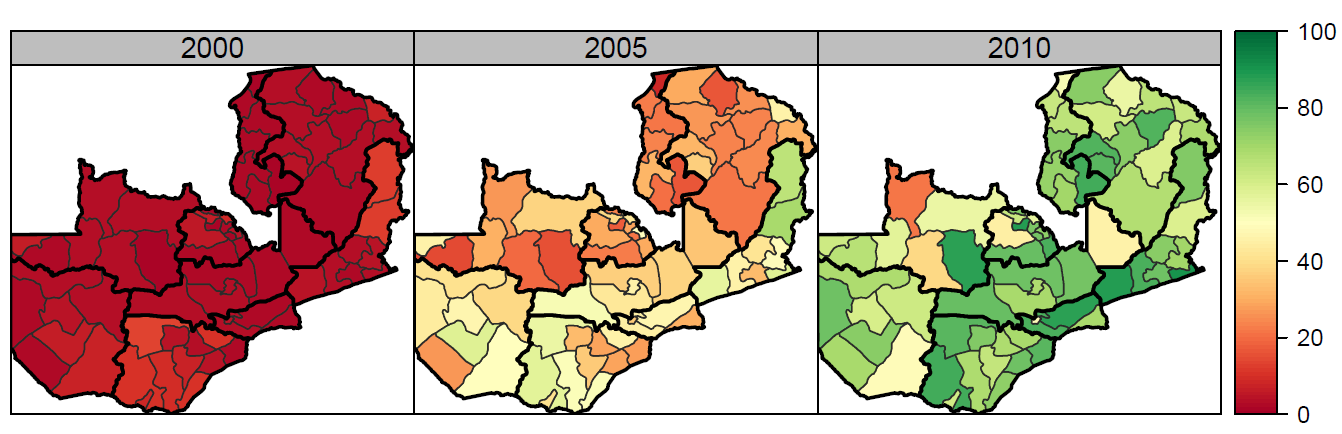
**

**Polio immunization (3 doses)**

**
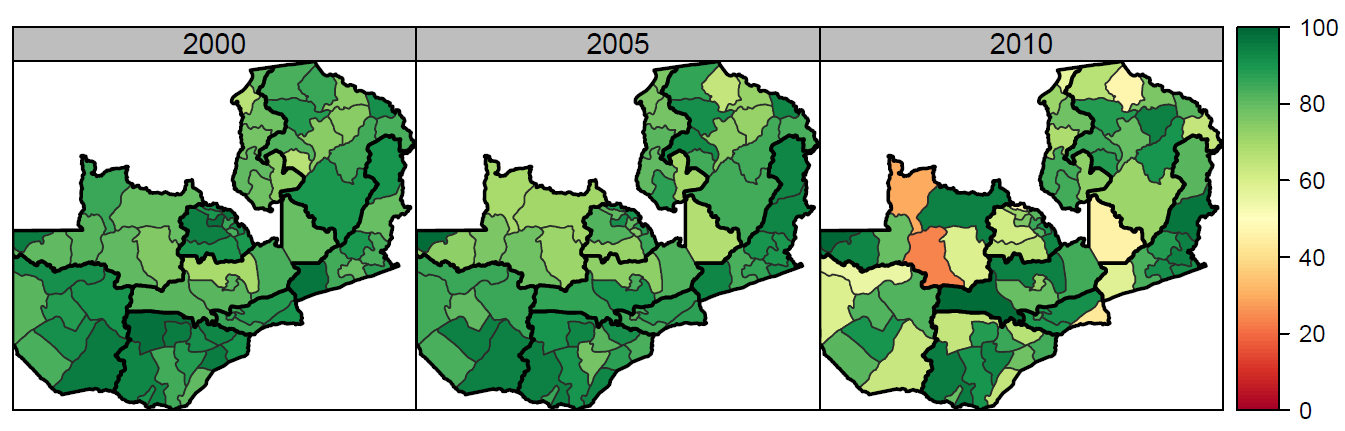
**

**Skilled birth attendance**

**
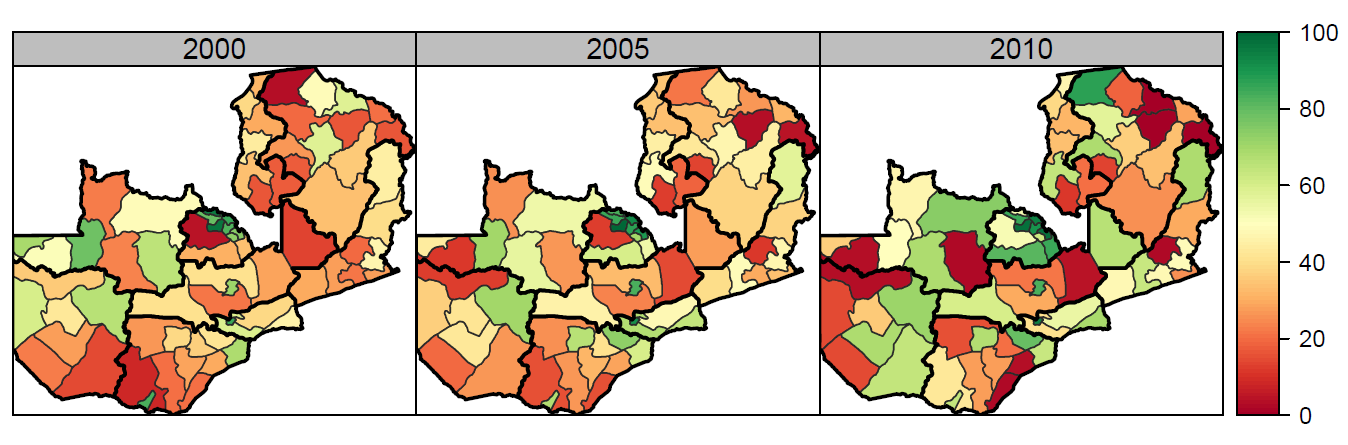
**

**Underweight**

**
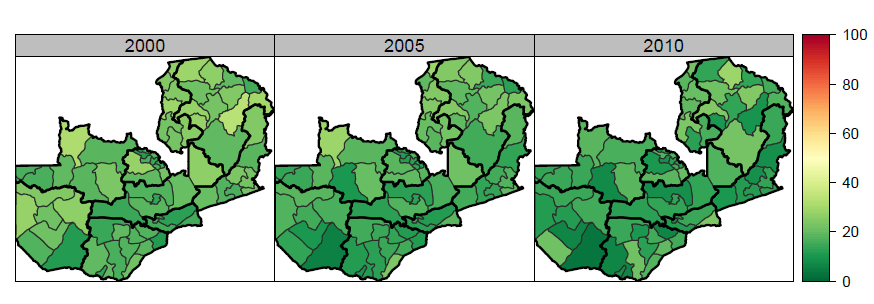
**
