# Supplementary material for: Benchmarking health system performance across districts in Zambia: a systematic analysis of levels and trends in key maternal and child health interventions from 1990 to 2010
Source: BMC Med. 2015 Apr 2;13:69. doi: 10.1186/s12916-015-0308-5 (PMC4382853; doi:10.1186/s12916-015-0308-5)
Supplement: Additional file 3: — features graphics accompanied by a scale that represents intervention coverage or the proportion of children who were underweight ranging from 0% to 100%. [file 12916_2015_308_MOESM3_ESM.docx]

| **Data source** | **Years represented** |
| --- | --- |
| **Surveys** | |
| Demographic and Health Survey (DHS) | 1992, 1996-1997, 2001-2002, 2007 |
| Malaria Indicator Survey (MIS) | 2006, 2008, 2010, 2012 |
| Multiple Indicator Cluster Survey (MICS) | 1999 |
| Living Conditions Monitoring Survey (LCMS) | 1996, 1998, 2002-2003, 2004-2005, 2006, 2010 |
| Health Facility Census | Japan International Cooperation Agency (JICA) (2005-2006) |
| Sexual Behavior Survey (SBS) | 2005, 2009 |
| Household Health Coverage Survey | 2008 |
| Netmark Survey reports | 2000, 2004 |
| **Population Censuses** | |
| National census | 1990, 2000, 2010 |
| **Administrative Sources** | |
| Health Management Information System (HMIS) | 2000-2008; 2009 |
| Malaria intervention databases | National Malaria Control Centre (NMCC) (2005-2010) |
| Facility-level PMTCT services | National AIDS Council quarterly status report (2005-2009) |
| HIV/AIDS projections | Central Statistical Office (CSO) (2005) |
| Drug supply and delivery records | Medical Stores Limited (MSL) (2007-2010) |
| Precipitation data | Global Precipitation Climatology Centre (1986-2012) |
| Malaria endemicity (*PfPR_2-10_*) | Malaria Atlas Project (2007, 2010) |
